# Dimension of visual information interacts with working memory in monkeys and humans

**DOI:** 10.1038/s41598-022-09367-7

**Published:** 2022-03-29

**Authors:** Daniel J. Fehring, Alexander J. Pascoe, Zakia Z. Haque, Ranshikha Samandra, Seiichirou Yokoo, Hiroshi Abe, Marcello G. P. Rosa, Keiji Tanaka, Tetsuo Yamamori, Farshad A. Mansouri

**Affiliations:** 1 Cognitive Neuroscience Laboratory, Department of Physiology, Monash Biomedicine Discovery Institute, Monash University, 26 Innovation Walk, Clayton, VIC 3800 Australia; 2 ARC Centre of Excellence in Integrative Brain Function, Monash University, Clayton, VIC Australia; 3 Molecular Analysis of Higher Brain Function, RIKEN Center for Brain Science, Wako, Saitama Japan; 4 Department of Physiology, Monash Biomedicine Discovery Institute, Monash University, Clayton, VIC Australia; 5 RIKEN Center for Brain Science, Wako, Saitama Japan

**Keywords:** Neuroscience, Cognitive neuroscience

## Abstract

Humans demonstrate behavioural advantages (biases) towards particular dimensions (colour or shape of visual objects), but such biases are significantly altered in neuropsychological disorders. Recent studies have shown that lesions in the prefrontal cortex do not abolish dimensional biases, and therefore suggest that such biases might not depend on top-down prefrontal-mediated attention and instead emerge as bottom-up processing advantages. We hypothesised that if dimensional biases merely emerge from an enhancement of object features, the presence of visual objects would be necessary for the manifestation of dimensional biases. In a specifically-designed working memory task, in which macaque monkeys and humans performed matching based on the object memory rather than the actual object, we found significant dimensional biases in both species, which appeared as a shorter response time and higher accuracy in the preferred dimension (colour and shape dimension in humans and monkeys, respectively). Moreover, the mnemonic demands of the task influenced the magnitude of dimensional bias. Our findings in two primate species indicate that the dichotomy of top-down and bottom-up processing does not fully explain the emergence of dimensional biases. Instead, dimensional biases may emerge when processed information regarding visual object features interact with mnemonic and executive functions to guide goal-directed behaviour.

## Introduction

Cognitive control enables the flexible use of limited cognitive resources to achieve goals^1–5^. In a changing environment, there are often more visual stimuli, with various features, than can be actively processed at any given time^6^. Therefore, cognitive control may be facilitated by detecting relevant stimulus features or dimensions (such as colour, shape, or movement) and suppressing irrelevant features/dimensions to achieve goals^2,4,7–11^. Working memory is of particular importance in these processes, as it facilitates the online maintenance and updating of goal-relevant information for guiding behaviour^12^.

In certain tasks that require a decision based on dimensional information, e. g. matching based on the colour or shape of visual objects, humans demonstrate a behavioural advantage (bias), in allocating attention to and redirecting attention away from particular visual dimensions^13–19^. Such dimensional biases are observable in children and influence their performance in cognitive tasks^20^ and saccadic eye movements^21^. However, these dimensional biases are not present in children with autism spectrum or other neuropsychological disorders, suggesting an association between dimensional biases and the pathophysiological and developmental changes in the involved brain networks^13,15–17,19,21,22^. Therefore, understanding such biases and their underlying neural mechanisms might bring crucial insights to the neuropathological bases of neurodevelopmental and neuropsychological disorders. However, it is still unclear where and how these dimensional biases emerge in the human brain, and their link to cognitive impairments in neuropsychological disorders remain unknown.

The Wisconsin Card Sorting Test (WCST) is a commonly used neuropsychological test to assess cognitive flexibility in shifting between abstract dimensions/rules^1,12–14,23–27^. In the WCST, the information of the currently relevant dimension for matching should be kept in working memory to guide the dimension-based behaviour. However, as the sample and matching test items are displayed concurrently, information of object features (colour and shape) are available in the sample presentation period and at the time of matching. In a computerised version of the WCST, we found that humans showed a significant bias to colour dimension, while monkeys performing the WCST with the same visual objects showed a significant bias to shape dimension^13^. These behavioural biases manifested as shorter response times and higher accuracy in trials which required matching in the preferred dimension^13^. Moreover, dimensional bias also influenced event-related arousal levels in humans (indexed by electrodermal activity). Task-related events evoked a smaller electrodermal response when colour-matching than when shape matching, indicating that dimensional biases have the capacity to modulate both cognitive and autonomic processes^13^.

Two independent scenarios may explain the presence of dimensional biases to colour in humans and to shape in monkeys. The first scenario, which we will consider as ‘top-down processes’, assumes that attentional modulations influence neural networks involved in the processing and integration of visual information with more advanced cognitive functions. Such attentional modulations, presumably mediated by the brain's attention network (including prefrontal cortical regions, anterior cingulate and parietal cortices)^13,26,27–34^, enhance information processing for a particular dimension and concurrently inhibit the processing for the other non-preferred dimension, and may be guided by working memory content^12,14^. In this scenario, the dimensional bias originates from an inherent or learned advantage in attentional modulation of processing and integrating colour or shape information with other cognitive functions.

In contrast, the second scenario, which we will consider as ‘bottom-up processes’, proposes that dimensional bias merely originates from advantageous processing of sensory information regarding a particular object feature^13^. This alternative explanation assumes that 'hardwired' or experience-dependent processing advantages for a particular object feature (shape or colour) emerge in the early visual pathways (even retina) and consequently enhance the detection and matching of the objects. Such a processing advantage would be independent of task demands and involved cognitive functions, and therefore will lead to a behavioural bias to a particular dimension whenever information of colour or shape is used.

Our recent study^13^ showed that selective lesions in different prefrontal cortical regions did not abolish the bias towards shape in monkeys, suggesting that the bias was not dependent on prefrontal-mediated top-down attentional modulation. Thus, these findings support the second scenario in which the bias originates in earlier stages of colour or shape information processing and enhances the detection and matching of visual objects in a particular dimension. In a follow-up study^7^, we showed that the dimensional bias to shape in monkeys could also be seen in the context of the Stop-signal task, manifested as an enhanced inhibition ability when a change in shape dimension (compared to a change in colour dimension) was used to trigger response inhibition. These findings indicated that the dimensional bias was not limited to the context of the WCST (dimension-shifting tasks) and could also modulate other aspects of cognitive control, such as inhibition ability^7^. These observations further supported the above-mentioned second scenario, in which the dimensional biases emerge from earlier processing advantages that influence various cognitive functions. Therefore, in the dichotomy of the top-down and bottom-up modulation^13,33,35,36^ of information processing; some previous findings^7,13^ suggest that the dimensional bias might be mediated through processing advantages in the earlier stages of visual information processing (bottom-up processes), independent of higher-level cognitive functions (such as set-shifting or response inhibition).

Previously, in a modified version of the WCST, we altered the level of conflict between the two dimensions (colour and shape) in a trial-by-trial manner and therefore introduced two randomly intermingled trial types (congruent and incongruent)^13^. We found that, in both species, the dimensional biases were influenced by conflict level, suggesting that dimensional biases were not necessarily independent of task demands or cognitive processes involved in the detection and resolution of conflict. Thus, it is still unclear whether the dimensional bias merely depends on the bottom-up or top-down processing advantages.

**Figure 1:**
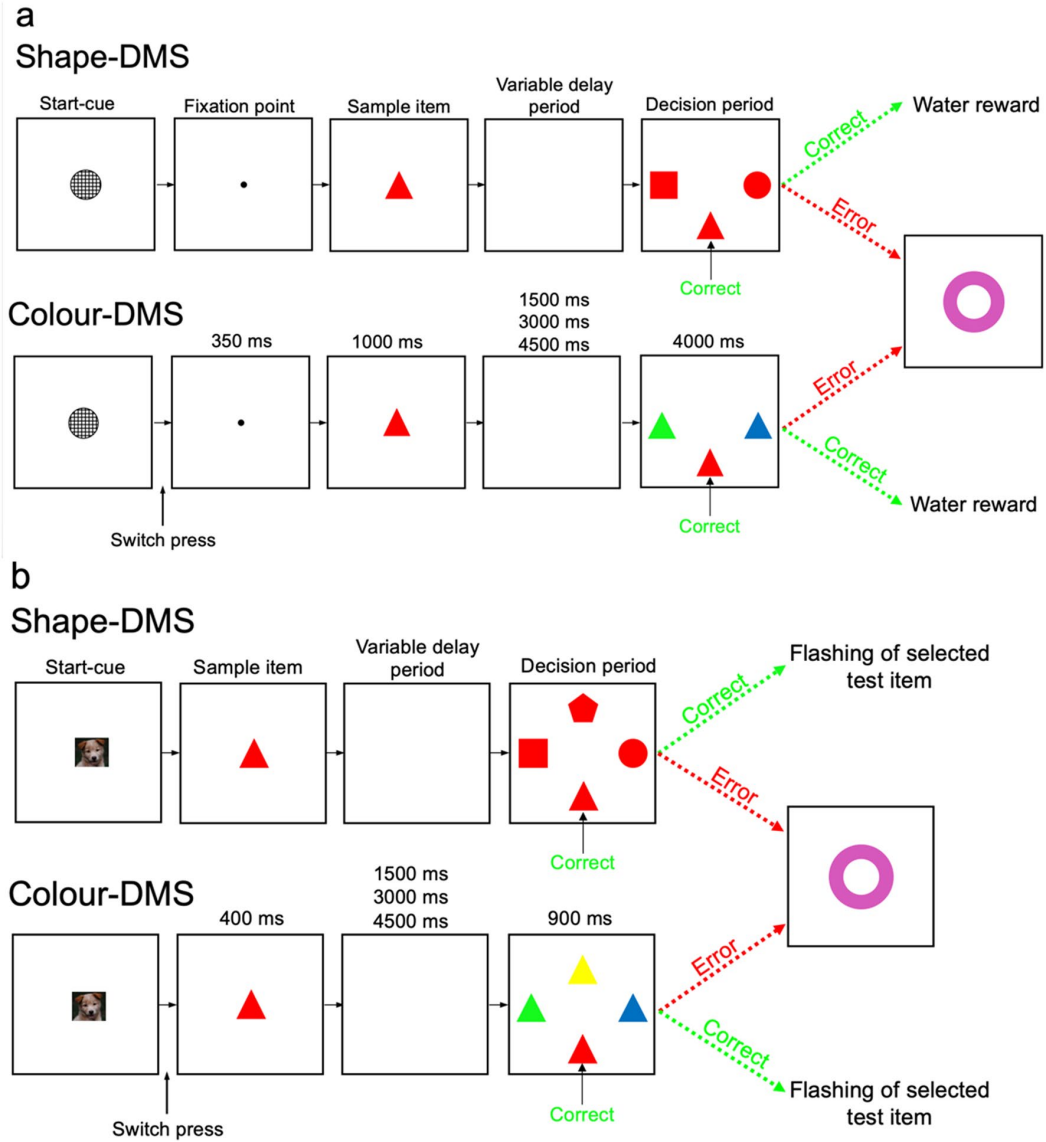
Computerised delayed matching to sample task. (**a**) In the DMS used for testing monkeys, each trial commenced by the appearance of a start-cue (an image), and subjects pressed and held a switch. Pressing the switch changed the start-cue to a fixation point, which was replaced by a sample (coloured shape) if the switch was still held. The sample was shown for 1000 ms before disappearing. Following a delay, the four test items appeared. The delay period randomly varied between 1500 ms, 3000 ms, and 4500 ms. If the correct matching item (target) was selected, feedback was provided (the target item flashed off (for 200 ms) and on (for 200 ms). The selection of the wrong target, early switch release, or failure to respond within the time window (4000 ms) were erroneous responses and led to the disappearance of all items, and an error signal was displayed for 500 ms (pink annulus). In shape-DMS trials, the test items had the same colour as the sample but differed in shape, and therefore the participants were required to match by shape. However, in colour-DMS trials, the test items had the same shape as the sample but differed in colour, and therefore the participants had to match by colour. Shape- and colour-DMS trials were intermingled. In the DMS used in monkeys, three test items were presented on the left, right and bottom of the screen during the matching period. In Experiment 1, the delay was fixed at 1500 ms, while in Experiment 2, the delay was randomly varied (1500 ms, 3000 ms, or 4500 ms). Upon selection of the correct target, the monkeys received a liquid reward (water). (**b**) In the DMS used in humans, the trial structure was similar, however, four test items were presented on the left, right, top and bottom of the screen during the matching period (900 ms).

In the current study, we used a specifically-designed task (Fig. 1) to assess the two aforementioned scenarios, to provide insight into the modulatory processes that underlie dimensional biases in monkeys and humans (towards shape and colour, respectively). Specifically, this task allowed us to examine whether dimensional biases are independent of higher-level processing of dimensional information. Furthermore, the inclusion of the variable delay enabled the examination of whether mnemonic aspects of the task, which potentially depend on higher-level cognitive processes^37,38^, could influence the dimensional bias in monkeys' behaviour. In addition, if the enhanced visual information processing and consequently enhanced detection and matching of object features (colour or shape) is the only mechanism mediating the behavioural bias to a particular dimension, then such a processing advantage would not occur in the absence of visual objects. Thus, if matching is required based on the memory of the object, rather than the actual visual information of the object, the bottom-up processing advantage might not occur, and therefore, dimensional biases would not manifest in monkeys' or humans' behaviour in this task.

We also studied humans' behaviour in the context of this modified version of the delayed matching to sample task (DMS) (Fig. 1b) to examine whether humans show dimensional bias in the context of this modified DMS, and whether the direction of bias is similar or different (as previously seen in the WCST^13^) between these two primate species.

## Methods

### Participants

#### Study in monkeys

Two adult macaque monkeys [Female *Macaca fuscata*; Animal 1 (6.7 kg) and Animal 2 (7.3 kg)] were included in this study. The subjects had no previous experience with any other task before learning the DMS. After each daily training–testing session, monkeys were returned to primate home cages in a 12 h light–dark cycle. Food access was ad libitum; however, water scheduling was employed to motivate the animals to perform the task, with the animals receiving their daily water during and after the sessions. The study was approved by the Research Ethics Committee of RIKEN Center for Brain Science. The study was conducted in accordance with relevant guidelines and regulations. Moreover, all methods are reported in accordance with ARRIVE guidelines.

#### Study in humans

Thirty-four (21 female) healthy participants between the ages of 20–38 years old (22.79 ± 0.56; mean ± standard error) were included in this study. Those participants with a history (diagnosed or self-reported) of neurological disorders were excluded from the study. Written informed consent was obtained from all participants before their involvement. The study was approved by the Human Research Ethics Committee of Monash University. The study was conducted in accordance with relevant guidelines and regulations. Moreover, the study was in accordance with The Code of Ethics of the World Medical Association (Declaration of Helsinki).

### Apparatus

#### Study in monkeys

Monkeys completed the task sitting in a primate chair facing a touchscreen (Elo Entuitive 15"), and a switch was positioned below the screen. To facilitate access to the touch screen, the front panel of the monkey chair was removed. All stimuli were shown on a black background on the touchscreen. Subjects performed the task in a well-ventilated and sound-attenuated room. During training and completion of the task, the monkeys received a water reward for each correct trial via a computer-controlled solenoid valve.

#### Study in humans

Participants were seated at a distance of approximately 60 cm from a 17″ touchscreen (3 M MicroTouch). A switch was also placed on the table in line with the base of the screen. The participants were observed via a monitoring camera to ensure that they adhered to the instructions. Prior to the first session, the participants were required to read a written explanation of the cognitive test procedure and also received pre-defined verbal instructions at the start of their testing session.

### Behavioural task

Both monkeys and humans performed a computerised version of the DMS (Fig. 1). We used the CORTEX program (National Institute of Mental Health) to conduct the experiment and data acquisition at millisecond resolution.

#### Studies in monkeys: Experiment 1

In each trial, a start-cue (grey circle) instructed monkeys to press and hold the switch. Once held, a fixation point appeared for 350 ms, and was then replaced by a sample item (coloured shape) positioned at the centre of the screen for 1000 ms (Fig. 1a). If the switch was still pressed, the sample disappeared and a delay period continued for 1500 ms when there was no item on the screen. A fixed delay period was implemented across all trials. At the end of the delay period, three test items were presented on the left, right, and bottom of the screen. Monkeys had to release the switch and touch the matching test item (target) within a limited time window (4000 ms from the onset of test items). In shape-DMS trials, all test items were the same colour as the sample but differed in shape, and therefore the monkeys had to use shape matching to find the target. However, in colour-DMS trials, all test items were the same shape as the sample but differed in colour, and therefore the monkeys had to use colour matching to find the target (Fig. 1a). Shape- and colour-DMS trials were intermingled and randomly presented. In total, there were 36 possible items created by combining six colours (red, green, blue, cyan, magenta and yellow) and six shapes (circle, square, triangle, cross, ellipse and hexagon). In each trial, the sample and test items were randomly selected from these 36 items considering the requirement for colour- or shape-DMS. There was no cue to indicate whether a trial required matching based on the colour or shape of the sample, and monkeys could notice the relevant dimension for matching only when the test items were presented. Therefore, in shape- and colour-DMS trials, monkeys had to memorise both the colour and shape of the sample during the delay period and use that information (colour or shape) when the test items were presented (Fig. 1a).

If the correct target was selected and touched, feedback was provided [the target item flashed off (for 200 ms) and on (for 200 ms)], and a liquid reward (water) was delivered for each correct trial via a computer-controlled solenoid valve. If an erroneous response (touching the non-matching item, early release of the switch, or failure to respond within the time window) occurred, all items disappeared, and an error signal was presented (Fig. 1a). Testing sessions commenced with a practice block where monkeys had to complete 9 successful trials in 10 consecutive trials to advance into the main block where data was collected. In each daily session, the data collection block continued until monkeys completed 300 trials. The monkeys' performance was assessed in 12 sessions in experiment 1, and 10 sessions in experiment 2.

Colour-DMS and shape-DMS trials were randomly intermingled in each session, and therefore the monkey could not predict which dimension would be relevant in each trial until the onset of the test items (start of the matching period). This task design required the monkey to maintain both colour and shape information of the sample during the delay period. In contrast to the WCST, in the modified version of the DMS, the sample was not presented in the matching period, and therefore the matching had to be done based on the maintained memory of the previously shown visual object (sample). We investigated whether the dimensional bias appeared in monkeys' behaviour when there was no sample (visual object) in the visual scene during matching. The absence of a dimensional bias in this context would imply that the emergence of such bias requires the presence of the object (sample) in the visual field to enable processing advantages in the early visual pathways.

##### Training

Monkeys were first trained to press a switch following the onset of a start-cue and touch the visual items on the screen to receive a reward. They were then trained in a version of the DMS in which one of the test items matched the sample (was identical), and the other test items differed from the sample in both shape and colour. This version of the DMS was easier than the final version because the distractor stimuli did not match the sample in either dimension. In this version of the DMS, sample and test items were selected from the same 36 visual item pool used in the final version of the DMS. After learning this version of the DMS, monkeys were trained with the final version of the DMS in which the two distractor test items matched the sample in one of the visual dimensions but differed in the other dimension, and thus the macaque had to use only one of the visual dimensions (colour or shape) for selecting the target item (Fig. 1a).

#### Study in monkeys: Experiment 2

In experiment 2, the task and contingencies were the same as in Experiment 1. Previous studies in macaque monkeys have shown that performance in working memory tasks is sensitive to the duration of the delay period (from sample disappearance to the onset of test items); presumably, because working memory processes are adversely affected by increases in the period for which the information must be transiently maintained^37–42^. These studies indicated that varied delay periods, randomly presented, at both 500 ms^39^ and 2000 ms^40^ influenced performance; monkeys' accuracy decreased as the delay period increased. However, such dissociated effects were not observed in delay periods longer than 8000 ms^40^. Therefore, to examine the influence of working memory load on the dimensional biases, in Experiment 2 the delay between the disappearance of the sample and the test items' onset was changed trial-by-trial to control the mnemonic demands of the task: the delay was randomly selected to be either 1500 ms, 3000 ms, or 4500 ms.

#### Study in humans: Experiment 3

At the beginning of each trial, a start-cue (an image) indicated to participants to press and hold down the switch (Fig. 1b). While held, a sample item appeared on the middle-centre of the screen for 400 ms. If the participant continued to hold down the switch in the trial, the sample item disappeared and a delay period continued for either 1500 ms, 3000 ms or 4500 ms with no visual stimulus on the screen. Previous studies in humans have indicated that it takes at least 6 s for the working memory load to influence performance in DMS tasks. Therefore, to detect the behavioural influence of variable delay periods, much longer delay period should be included in the task paradigm^43–45^. However, including such long delays cumulatively lengthens the testing session and might lead to distraction (mind wandering) or fatigue and introduce additional confound to detect dimensional bias. Therefore, we did not include long variable delays in the DMS for humans and instead, included shorter variable delays between the disappearance of the sample and the test items' onset to prevent any anticipatory (prepared) responses, which participants may deliver before or just after the onset of the test items. The delay period (1500 ms, 3000 ms or 4500 ms) was changed trial-by-trial. After the delay period, four test items appeared on the left, right, top and bottom of the screen, whereby the participants were required to release the switch and touch the matching test item (target) within a limited response window (900 ms from the appearance of the test items) (Fig. 1b). Four test items were used in the human study (Experiment 1), rather than the three test items used in the macaque studies (Experiment 2–3), as past studies contrasting performance of humans and monkeys in the DMS indicated that with 3 test items, 14-year-old children perform the task at ceiling^46^. Therefore, to avoid such ceiling effects (which may mask any observable influence of dimension), while maintaining an analogous task design, another test item was added in the human study to increase the difficulty.

Similar to the DMS task used for testing monkeys, both shape-DMS and colour-DMS trials were included, and participants were required to match based on the colour or shape of the sample (Fig. 1b). Shape- and colour-DMS trials were intermingled and randomly presented. In each trial, the sample and test items were randomly selected according to either shape or colour dimension. Participants were able to notice the relevant dimension for matching only after the test items were presented. If the participant selected the correctly matched test item, the test item would flash on the screen as feedback, and then all items would disappear before the subsequent trial commenced. If the incorrect test item was selected, or the participant failed to select a test item within the response window, an error signal (pink annulus) would appear on the screen (Fig. 1b). The same set of 36 visual items (used for testing monkeys) was used to test humans.

Participants were instructed to use only their dominant hand index finger to press and hold the switch and select the test item on the screen. In each testing session, participants had to achieve 100 correct trials to complete the task. In the study with humans, participants performed the DMS in three different background acoustic conditions (classical music, white noise and silence) in each daily session. Each of the acoustic conditions was played through headphones for each participant. The background acoustic condition did not have any significant effect on participants' performance in the DMS, therefore their effects are not reported in this manuscript.

### Electrodermal activity

We recorded human participants’ electrodermal activity (EDA), a measurement of skin conductance in microsiemens (μS), whilst they performed the DMS. To measure the event-related changes in EDA in real-time, the electrodermal recording unit software (ML116 GSR Amp, ADInstruments) was employed at a sampling rate of 75 kHz^47,48^. Two electrodes were attached to the index and ring fingers of the participants’ non-dominant hand to measure skin conductance. Since the electrodes are sensitive to movement-related noise, participants were advised to keep their hand still. We calculated the difference between the minimum and maximum amplitudes of the phasic activity for each event-related electrodermal signal within a 3 s time epoch following target selection^47,49^. However, due to various factors that included motion artefacts, cold hands, and low sweating levels, EDA readings were not adequately recorded for three participants. Two observers blinded to the data analyses assessed the quality of recorded EDA for exclusion of unreliable data. Therefore, EDA data obtained from 31 participants were used in EDA data analysis.

### Data analyses

The same analytical approach was used in all experiments. Repeated-measures Analysis of Variance (ANOVA) tests were used to assess the effects of matching dimension, and variable delay (in Experiment 2). Session means were used as data points, and therefore a Monkey factor was also included in the ANOVA. Response time was calculated as the time between the onset of test items and the release of the switch. Response time was then normalised by dividing each value by the grand average for all conditions in each individual. This normalisation procedure controls for the differences in mean response time between participants, while maintaining the relative difference between the conditions^4,13,31,47^. The EDA data in humans was also normalised by using a similar approach to control for the variability of participants' sweating levels. Accuracy was calculated as the percentage of correct responses in each testing session. All subjects had to respond within a response window (4000 ms for Monkeys and 900 ms for Humans), and all responses outside this window were considered a timeout error, and therefore all response time values in correct trials were within the response window. All behavioural data points were used in analyses without removing outliers, as the implementation of arbitrary procedures for removing outliers may bias results and subsequent statistical analysis. Mauchly's test was used to examine sphericity for all repeated-measure analyses, and Greenhouse–Geisser correction was applied when necessary. Within ANOVA, partial eta squared indicates the proportion of the total variance explained by the effect and was reported for each significant effect. Two-tailed t-tests with Bonferroni adjustment for multiple comparisons were used for pairwise comparisons.

For correlation analyses, bivariate correlation analyses were used to examine whether the magnitude of dimensional biases correlated with performance measures. To examine the magnitude of dimensional bias, for each individual (in humans) or across different sessions (in monkeys), a 'Dimensional Bias Index' (DBI) was calculated for each performance measure (accuracy and response time in silent sessions). The DBI was calculated as the difference between correct colour-DMS and shape-DMS trials for each performance measure (response time or accuracy). Normalised values were used for calculation of the index. For all analyses, including the one used for calculation of the DBI index, we have used a uniform normalisation procedure in which the index was normalised by the average accuracy or mean response time. Accuracy was normalised only in correlation analyses to ease DBI comparison between performance measures. All bivariate correlation p values were false discovery rate (FDR) adjusted^50^.

### Ethics approval

The study in monkeys was approved by the Research Ethics Committee of RIKEN Center for Brain Science. The study was conducted in accordance with relevant guidelines and regulations. Moreover, all methods are reported in accordance with ARRIVE guidelines. The study in humans was approved by the Human Research Ethics Committee of Monash University. The study was conducted in accordance with relevant guidelines and regulations. Moreover, the study was in accordance with The Code of Ethics of the World Medical Association (Declaration of Helsinki).

## Results

### Studies in monkeys: Experiment 1

#### Monkeys' performance differed depending on the relevant dimension

**Figure 2:**
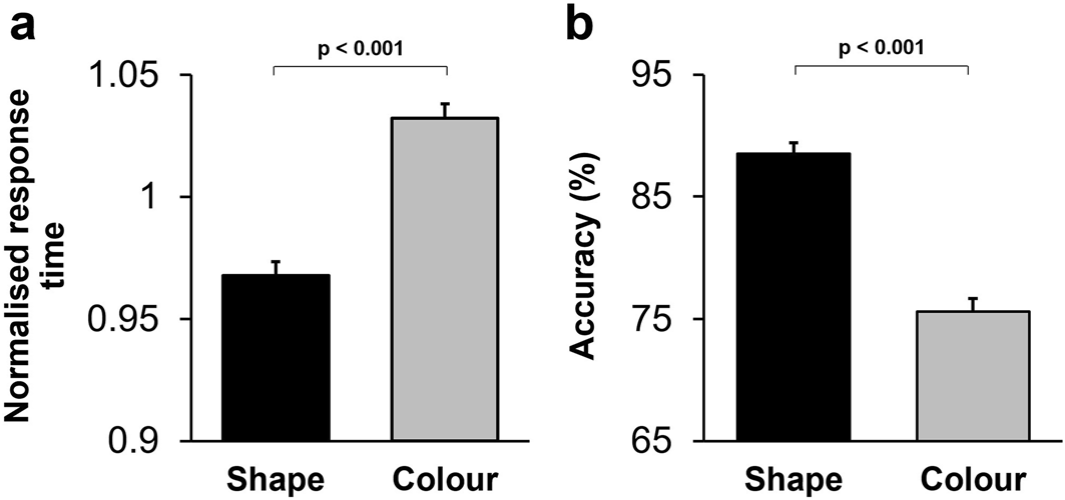
Dimensional bias influenced monkeys' performance in the delayed matching to sample task. (**a**) Mean normalised response time is shown for colour- and shape-DMS trials. Normalised response time was significantly shorter in trials where shape-matching was required than in trials where colour-matching was required. (**b**) The mean percentage of correct responses (accuracy) is shown for colour- and shape-DMS trials. Accuracy was significantly higher in shape-DMS trials compared to colour-DMS trials. In all Figures, error bars show the standard error, and p values indicate the significance level of the pairwise comparisons (with Bonferonni correction for multiple comparisons).

To assess whether the dimensional bias influenced monkeys' performance in the DMS we applied a two-way repeated-measures ANOVA [Dimension (colour/shape, within-subject factor) × Monkey (Animal 1/Animal 2, between-subject factor)] to the normalised response time in correct trials. There was a significant main effect of Dimension (F(1,22) = 30.36; *p* < 0.001) (Partial eta squared = 0.56): the response time was shorter in shape-DMS trials (Fig. 2a). The interaction between Dimension and Animal factor was not significant (F(1,22) = 0.41; *p* = 0.53), indicating that the behavioural advantage in shape-DMS trials (bias to shape dimension) was uniformly observed across monkeys. Moreover, we examined whether the difference in performance between colour-DMS and shape-DMS trials (dimensional bias) was significant in each animal by applying two-tailed paired t-tests (with Bonferroni correction for multiple comparisons) to the response time of correct trials in each monkey. The difference in performance between colour- and shape-DMS trials (shorter response time in shape dimension) was significant in both monkeys (for Animal 1: t_11_ = − 7.29; *p* = 0.001, and for Animal 2: t_11_ = − 2.68; *p* = 0.04).

We also examined the dimensional bias in monkeys' accuracy by applying the two-way repeated-measures ANOVA [Dimension (colour/shape, within-subject factor) × Monkey (Animal 1/Animal 2, between-subject factor)] to the mean percentage of correct trials in colour-DMS and shape-DMS trials. There was a highly significant main effect of Dimension (F(1,22) = 156.37; *p* < 0.001) (Partial eta squared = 0.87): monkeys' accuracy was significantly higher in trials that required matching based on shape dimension (compared to colour dimension) (Fig. 2b). The interaction between Dimension and Monkey was also significant (F(1,22) = 15.31; *p* = 0.001) (Partial eta squared = 0.41), indicating that although both monkeys displayed better performance when shape dimension was relevant, the magnitude of difference between colour-DMS and shape-DMS trials was different between the two monkeys. The main effect of Monkey was also significant (F(1,22) = 9.394; *p* < 0.01) (partial eta squared = 0.87): Animal 1 had higher accuracy than Animal 2 (84.68% ± 1.215 (mean ± standard error), and 79.41% ± 1.215, respectively). We further confirmed that the advantage in matching based on shape dimension was significant in each monkey by applying two-tailed paired t-tests (with Bonferroni correction for multiple comparisons) to the mean accuracy, which showed a significant difference between colour- and shape-DMS trials in both monkeys (for Animal 1: t_11_ = -7.30; *p* = 0.001, and for Animal 2: t_11_ = − 2.69; *p* = 0.04).

### Studies in monkeys: Experiment 2

In contrast to Experiment 1, where the delay between the disappearance of the target item and the appearance of the matching stimuli was fixed at 1500 ms, in Experiment 2 the delay period was randomly changed trial-by-trial (1500 ms, 3000 ms, or 4500 ms). Therefore, a Delay factor (within-subject factor) with three levels (1500 ms/3000 ms/4500 ms) was added to the ANOVA.

#### The dimensional bias in monkeys was dependent on the working memory load

Previous studies in macaque monkeys have shown that performance in working memory tasks is sensitive to the duration of the delay period (from sample disappearance to the onset of test items); presumably, because working memory processes are adversely affected by increases in the period for which the information must be transiently maintained^37,38^. Therefore, we introduced three different delay periods, in randomly intermingled trials, to change the difficulty of the DMS task for the monkeys. This allowed for the assessment of interactions between working memory load (task difficulty) and dimensional bias in monkeys.

**Figure 3:**
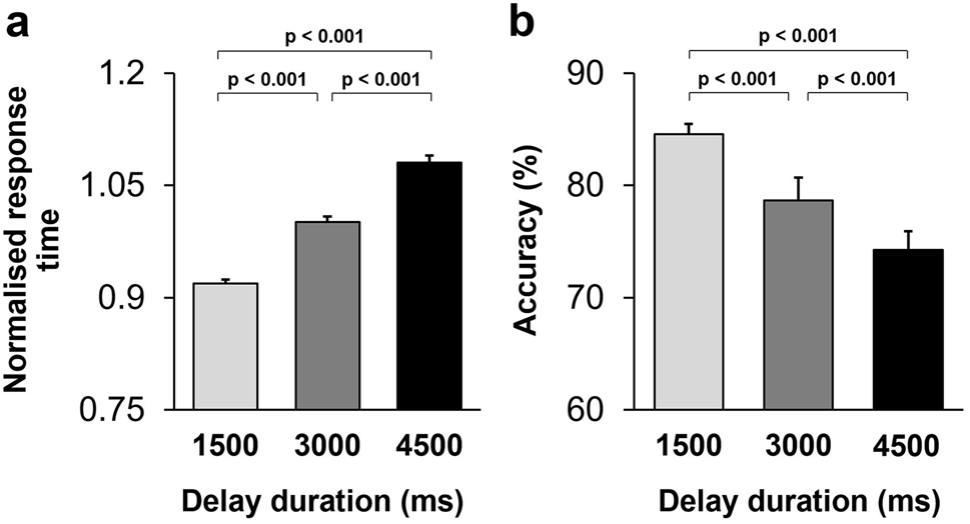
Monkeys' performance was modulated by the delay duration. (**a**) Mean normalised response time is shown for each delay duration. Monkeys' response time increased as the working memory load (delay duration) increased. (**b**) Mean accuracy is shown for the three delay durations. Monkeys' accuracy decreased as the working memory load (delay duration) increased.

A three-way repeated-measures ANOVA [Delay (1500 ms/3000 ms/4500 ms, within-subject factor) × Dimension (colour/shape, within-subject factor) × Monkey (Animal 1/Animal 2, between-subject factor)] applied to the normalised response time in correct trials revealed a highly significant main effect of Delay (F(2,36) = 73.01; *p* < 0.001) (Partial eta squared = 0.80): response time for target selection became longer as the delay period increased (Fig. 3a). Spearman's rho correlation coefficient was calculated to assess the relationship between Delay (1500 ms/3000 ms/4500 ms) and normalised response time. There was a significant correlation between variable delay and normalised response time (r_s_ = 0.89, *p* < 0.001).

**Figure 4:**
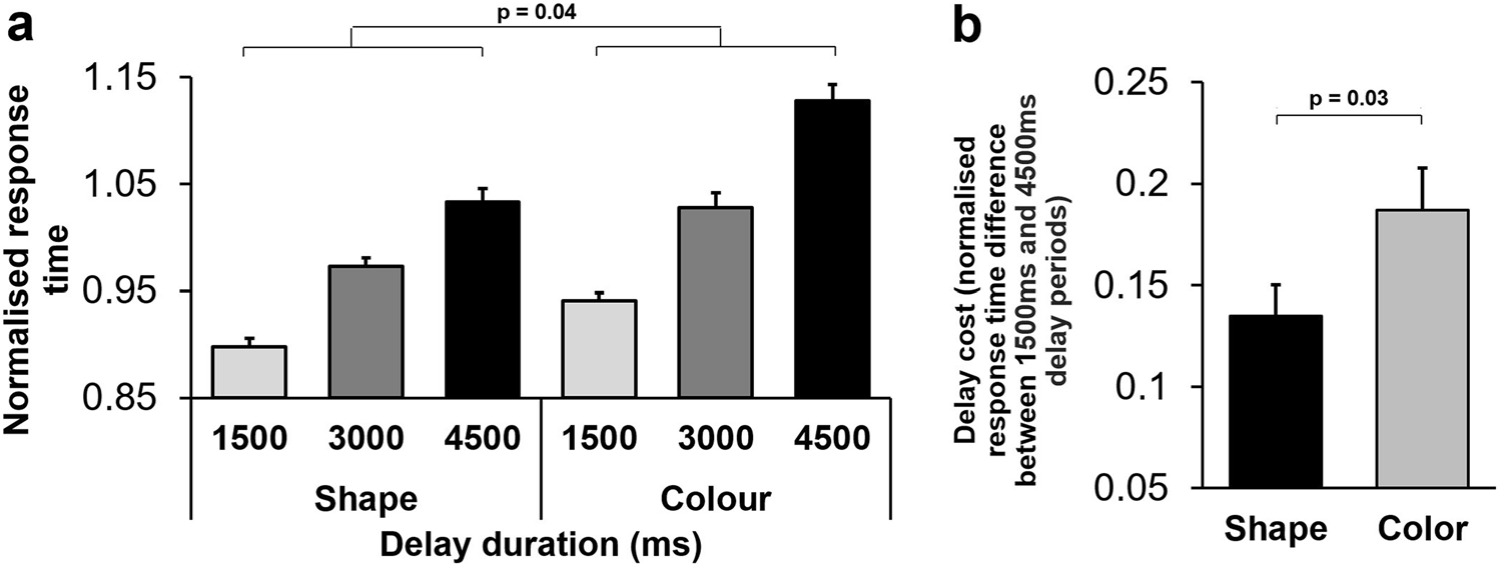
Delay duration and visual dimensions interactively modulated monkeys' response. time. (**a**) Mean normalised response time is shown for each delay duration in both matching dimensions. The increase in response time due to a longer delay duration was dependent on the matching dimension. (**b**) The magnitude of delay cost (normalised response time difference between the 1500 ms and 4500 ms delay period) is shown in both matching dimensions. The delay cost was higher in the colour dimension than in the preferred shape dimension.

The main effect of Dimension was also significant (F(1,18) = 28.47; *p* < 0.001) (Partial eta squared = 0.61). Importantly, there was a significant interaction between Delay and Dimension factors (F(2,36) = 3.49; *p* = 0.04) (Partial eta squared = 0.16), indicating that the dimensional bias was influenced by the task difficulty (working memory load). The difference in response time between delay durations was larger in the colour dimension than those differences in shape dimension (Fig. 4a,b). We also conducted Spearman's correlation analysis to assess the relationship between delay (1500 ms/3000 ms/4500 ms) and normalised response time in each dimension. There was a significant correlation between variable delay and normalised response time in each dimension (shape: r_s_ = 0.77, *p* < 0.001; colour: r_s_ = 0.79, *p* < 0.001). For ease of comparison, Fig. 4a presents the normalised response time for each delay duration in both matching dimensions, while 4b presents the magnitude of the delay cost (the response time difference between the 1500 ms and the 4500 ms delay period) for colour and shape dimensions. Post-hoc analyses (paired t-test) confirmed the difference in delay cost between colour and shape dimensions was significant (t(19) = − 2.3, *p* = 0.03; Fig. 4b). The significant interaction between the dimensional biases and the task difficulty suggests that the dimensional bias was not independent of the mnemonic demands of the task, which depend on more advanced stages of information processing.

#### Working memory load influenced monkeys' accuracy

To further examine the influence of working memory load on the percentage of correct responses in each dimension, a three-way repeated-measures ANOVA [Delay (1500 ms/3000 ms/4500 ms delay, within-subject factor) × Dimension (colour/shape, within-subject factor) × Monkey (Animal 1/Animal 2, between-subject factor)] was applied to monkeys' accuracy. The main effect of Delay was significant (F(2,36) = 11.67; *p* < 0.001) (partial eta squared = 0.39): an increase in the delay duration adversely affected monkeys’ performance (Fig. 3b). Spearman's rho correlation coefficient was calculated to assess the relationship between delay (1500 ms/3000 ms/4500 ms) and accuracy. There was a significant correlation between variable delay and accuracy (r_s_ = − 0.56, *p* < 0.001).

The main effect of Dimension was highly significant (F(1,18) = 101.58; *p* < 0.001) (partial eta squared = 0.85). The interaction of dimension and delay (F(2,36) = 0.75; *p* = 0.48) was not significant, indicating that difference in accuracy between various levels of working memory load (delay duration) was uniform between matching dimensions. We also conducted Spearman's correlation analysis to assess the relationship between Delay (1500 ms/3000 ms/4500 ms) and accuracy in each dimension. There was a significant correlation between variable delay and accuracy in each dimension (shape: r_s_ = − 0.37, *p* < 0.01; colour: r_s_ = − 0.46, *p* < 0.001). Like Experiment 1, there was a significant interaction between dimension and animal (F(1,18) = 5.61; *p* = 0.03) (partial eta squared = 0.24), where accuracy was higher in shape-DMS trials in both animals, but the magnitude of this bias was stronger in one of the animals (Animal 2).

### Study in humans: Experiment 3

In order to examine whether humans also display dimensional biases in the modified DMS task, and to compare the direction of any bias to those in monkeys, a multifactorial repeated-measures ANOVA was applied to the normalised response time in correct trials, percentage of correct responses (accuracy), and normalised EDA in correct trials, separately. The ANOVA included stage (first/second/third session) and dimension (colour/shape) as within-subject factors, and sex (female/male) as a between-subject factor.

#### Humans' performance differed depending on the relevant dimension

**Figure 5:**
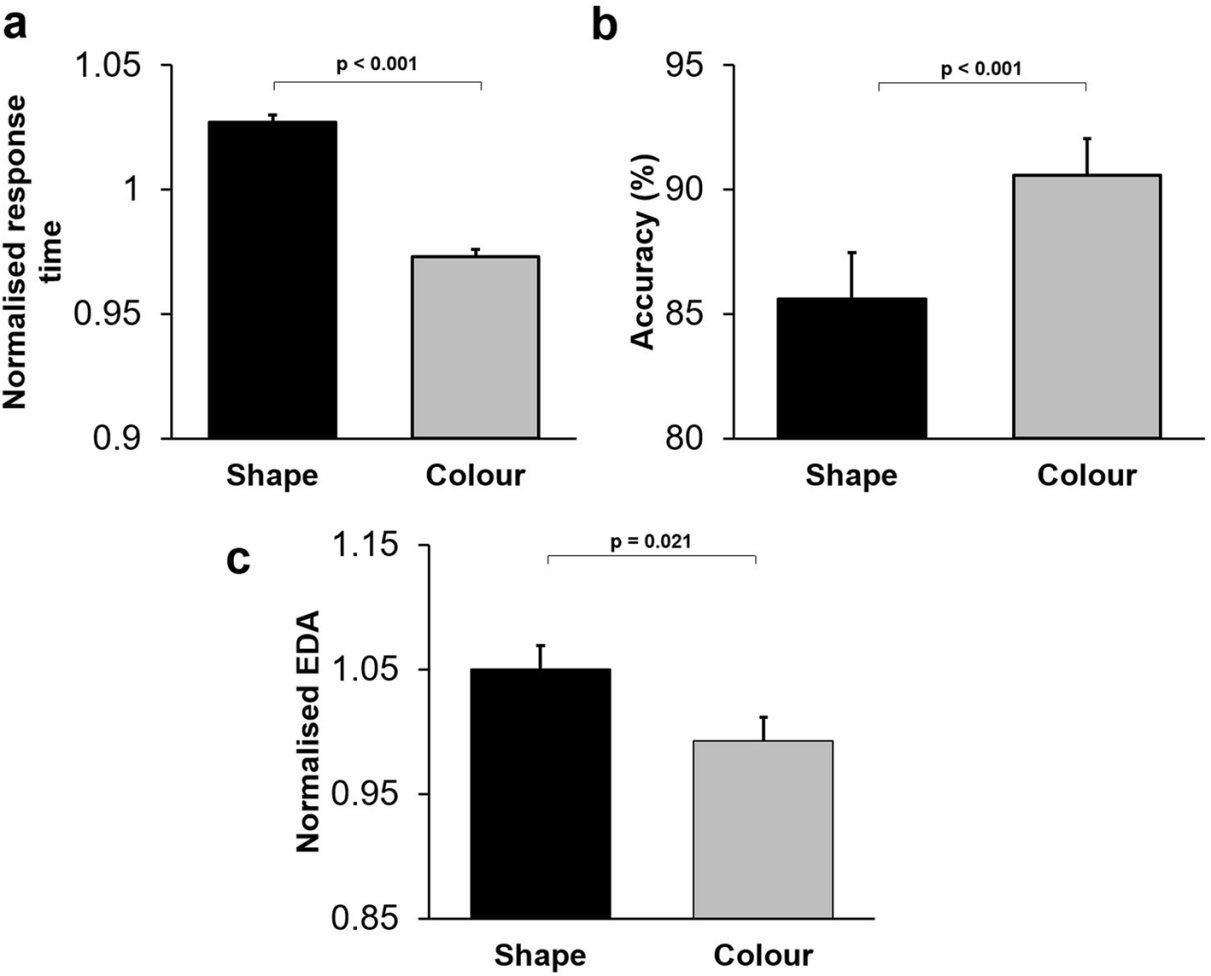
Dimensional bias influenced humans' performance in the delayed matching to sample task. (**a**) Mean normalised response time is shown for colour-DMS and shape-DMS trials. Normalised response time was significantly shorter in trials when colour-matching was required than in trials when shape-matching was required. (**b**) Mean accuracy is shown for colour-DMS and shape-DMS trials. Accuracy was significantly higher in colour-DMS trials compared to shape-DMS trials. (**c**) Mean normalised amplitude of electrodermal activity (EDA) is shown for colour-DMS and shape-DMS trials. EDA was significantly lower in colour-DMS trials compared to shape-DMS trials.

A three-way repeated-measures ANOVA [stage (first/second/third session, within-subject factor) × dimension (colour/shape, within-subject factor) × sex (female/male, between-subject factor)] was applied to the normalised response time in correct trials. The main effect of dimension (F(1,33) = 96.78; *p* < 0.001) (partial eta squared = 0.76) was highly significant: response time was shorter in the colour-DMS trials (Fig. 5a). The main effect of stage was not significant (F(2,66) = 0.63; *p* = 0.54): response time did not change across testing stages.

Additionally, we applied a three-way repeated measure ANOVA [Stage (first/second/third session, within-subject factor) × dimension (colour/shape, within-subject factor) × sex (female/male, between-subject factor)] to the mean percentage of correct trials in colour-DMS and shape-DMS trials. The main effect of dimension (F(1,33) = 42.31; *p* < 0.001) (partial eta squared = 0.58) was significant: accuracy was significantly higher in colour-DMS trials (Fig. 5b). The main effect of Stage was also significant (F(2,66) = 16.58; *p* < 0.001) (partial eta squared = 0.35): participants’ accuracy increased over the three stages of testing (within-session learning).

#### Humans' event-related electrodermal activity (EDA) differed depending on the relevant dimension

Event-related alterations in EDA reflects the changes in sympathetic nervous discharge and alterations in arousal-emotional levels. To assess whether the relevant dimension influenced participants' arousal-emotional state, we applied a three-way repeated measure ANOVA [stage (first/second/third session, within-subject factor) × dimension (colour/shape, within-subject factor) × sex (female/male, between-subject factor)] to the normalised EDA in correct trials. There was a significant main effect of dimension (F(1,31) = 5.93; *p* = 0.021) (partial eta squared = 0.17): EDA was lower in colour-DMS trials (Fig. 5c), suggesting that arousal-emotional level was lower when participants matched according to the preferred dimension. The main effect of Stage was not significant (F(2,62) = 2.85; *p* = 0.066).

#### The direction of dimensional biases differed between monkeys and humans

**Figure 6:**
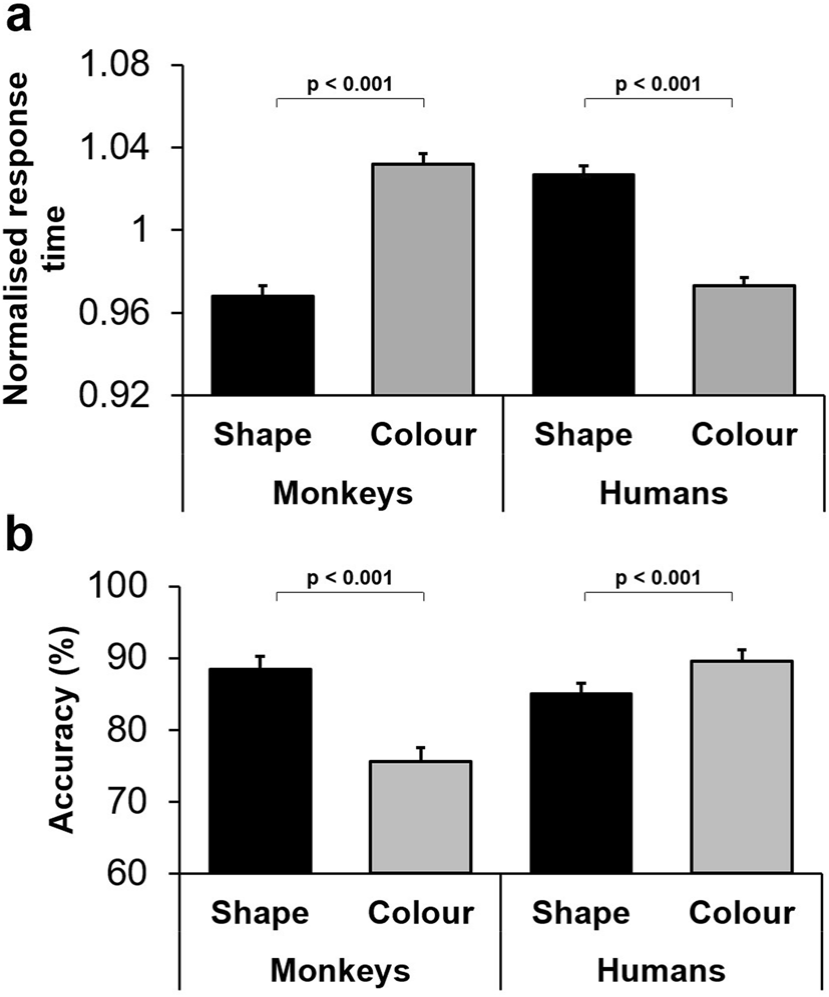
The direction of dimensional biases differed between humans and monkeys in the delayed matching to sample task. (**a**) Mean normalised response time is shown for colour- and shape-DMS trials for both primate species. In monkeys, the normalised response time was significantly shorter in shape-DMS trials than in colour-DMS trials. However, in humans, the opposite pattern was observed, with the normalised response time being significantly shorter in colour-DMS trials than in shape-DMS trials. (**b**) Mean accuracy is shown for colour-DMS and shape-DMS trials. Similar to response time, monkeys and humans displayed opposing biases in their performance, where monkeys' accuracy levels were significantly higher in shape-DMS trials, and humans' accuracy levels were significantly higher in colour-DMS trials.

Given the significant dimensional biases in both humans and monkeys in the DMS, we directly compared humans' and monkeys' normalised response times between colour- and shape-DMS trials. A two-way repeated-measures ANOVA [species (monkeys/humans, between-subject factor) × dimension (colour/shape, within-subject factor)] was applied to the normalised response time in correct trials. For this analysis, we used humans' performance data only from the Silent condition because monkeys also performed the DMS task in a silent condition (no background acoustic stimuli). As expected, the main effect of dimension was not significant (F(1,55) = 0.61; *p* = 0.44), because the dimensional biases were in opposite directions in humans and monkeys. However, there was a significant interaction between dimension and species (F(1,55) = 91.77; *p* < 0.001) (partial eta squared = 0.63), indicating that the direction of dimensional bias was significantly different between monkeys and humans (a bias to colour in humans, but a bias to shape in monkeys) (Fig. 6a).

We also compared accuracy between species by applying a two-way repeated-measures ANOVA [species (monkeys/humans, between-subject factor) × dimension (colour/shape, within-subject factor)] to the mean percentage of correct trials in colour-DMS and shape-DMS trials. The interaction between dimension and species was highly significant (F(1,55) = 115.94; *p* = 0.001) (partial eta squared = 0.68), indicating that the direction of dimensional bias differed between monkeys and humans (Fig. 6b).

#### The magnitude of dimensional biases correlated with performance

To further examine how dimensional biases influence behaviour, we examined the correlation between the magnitude of the dimensional biases and performance across individuals (in human participants) or across different sessions (in monkeys). A ‘Dimensional Bias Index’ was calculated as the difference between normalised performance in correct colour- and shape-DMS trials for each behavioural measure (response time and accuracy) in each participant or monkey. The DBI represents the magnitude of the dimensional bias toward a particular dimension (positive and negative values indicate a bias toward colour and shape, respectively): the further from zero, the stronger the bias toward the relevant dimension (zero indicates no dimensional bias).

**Figure 7:**
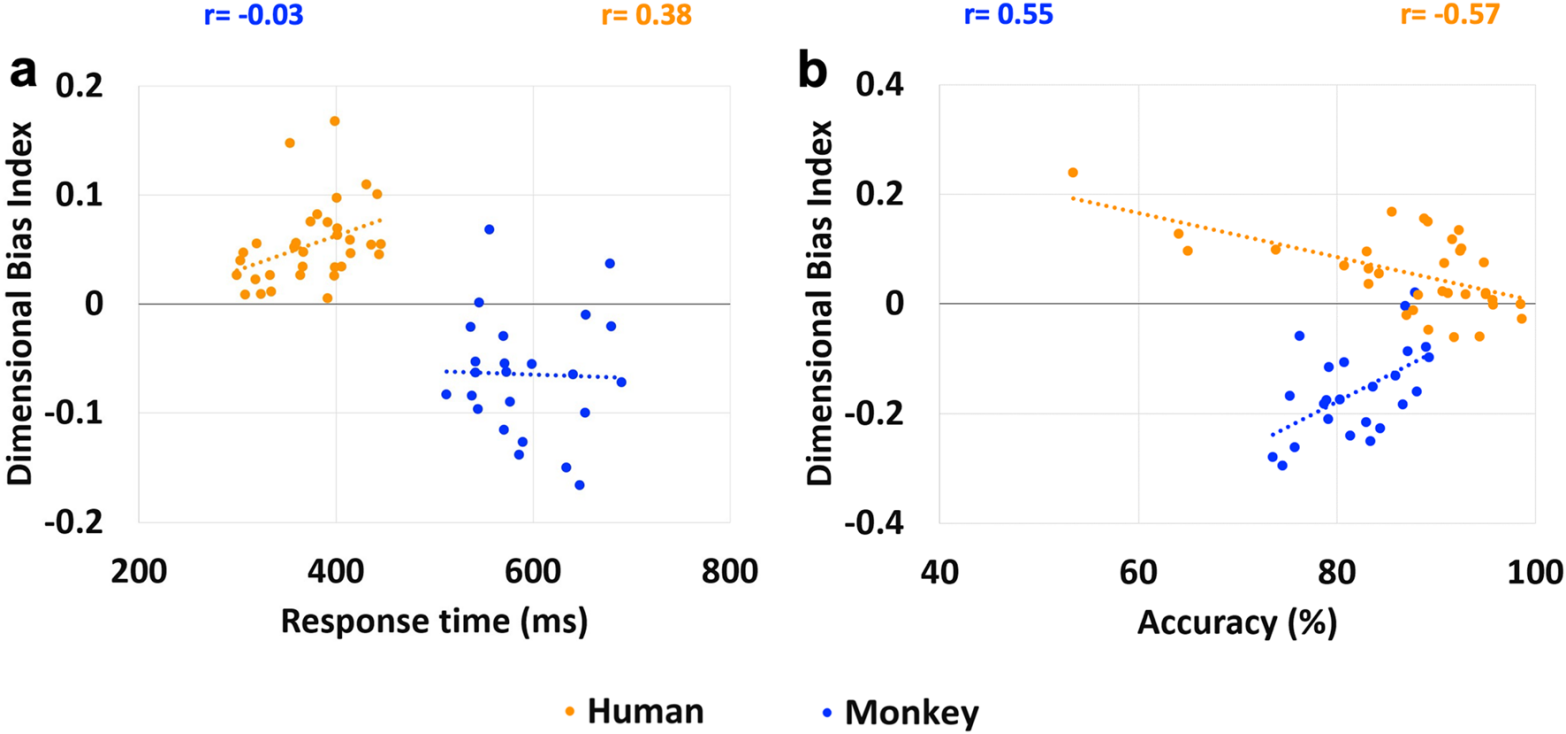
Correlation of the strength of dimensional biases and performance. (**a**) Correlation between the Dimensional Bias Index (DBI) and response time (ms) is shown for humans and monkeys. (**b**) Correlation between the DBI and the mean percentage of correct responses (accuracy) is shown for humans and monkeys. The dotted line indicates the fitted regression line. Each dot indicates the DBI for each human participant or each testing session (monkeys). The DBI represents the magnitude of bias towards a particular dimension (positive and negative values indicate a bias toward colour and shape, respectively). The further from zero (indicating no dimensional bias), the stronger the bias toward the relevant dimension. Note that the direction of dimensional bias differs between humans and monkeys.

The correlation between DBI and response time was significant in humans (r(33) = 0.38, *p* = 0.04); indicating that there was a positive correlation between the magnitude of dimensional bias and response time (Fig. 7a). However, in monkeys, the correlation between DBI and response time was not significant (r(24) = − 0.03, *p* = 0.90) (Fig. 7a).

There was a significant correlation between DBI and the mean percentage of correct trials (accuracy) in both humans (r(33) = − 0.57, *p* = 0.002) and monkeys (r(24) = 0.55, *p* = 0.01) (Fig. 7b). In both species, the magnitude of dimensional bias and accuracy were negatively correlated: i.e. if the magnitude of the dimensional bias (colour in human; shape in monkey) was higher, the accuracy was lower.

## Discussion

In a modified version of a working memory task, where matching had to be done based on the memory of visual objects, we found that macaque monkeys and humans showed significant behavioural advantage (bias) toward shape and colour dimensions, respectively (Fig. 6). The dimensional bias in monkeys interacted with the mnemonic demands of the task and appeared as better performance with the preferred dimension (Fig. 4). The dimensional bias in humans not only influenced performance, but also modulated the event-related arousal and autonomic nervous functions (Fig. 5c).

### Evidence in support of bottom-up processes as the origin of dimensional biases

In a recent study^13^, we have shown that selective and bilateral lesions in different prefrontal cortical regions, such as dorsolateral prefrontal cortex (DLPFC), anterior cingulate cortex (ACC), orbitofrontal cortex (OFC), frontopolar cortex or posterior cingulate cortex, do not abolish dimensional biases in monkeys performing the WCST. Although lesions in the DLPFC, ACC or OFC impaired monkeys' cognitive flexibility in shifting between rules, the behavioural bias to shape was not abolished^13^. These findings indicate that lesions in main nodes of the attention network such as the DLPFC and ACC could not eliminate the dimensional bias and therefore suggest that dimensional biases do not depend on prefrontal-mediated attentional modulation.

Further support for this assumption came from another recent study in monkeys^7^, which demonstrated that dimensional biases emerge in the context of a Stop-signal task, where the signal for response inhibition was delivered in either the colour or shape dimension. Monkeys consistently showed a better inhibition ability when the necessity for inhibition was signalled by a change in the shape dimension, than when signalled in the colour dimension^7^. Thus, in both the WCST^13^ and Stop-signal task^7^, monkeys demonstrate a behavioural bias toward the shape dimension, indicating that the emergence of dimensional bias is independent of the cognitive task and the involved cognitive functions. This suggests the bias might originate in the earlier processing stages and uniformly influence various cognitive functions that use colour or shape information for guiding behaviour. A dimensional bias to colour has also been shown in humans in the context of various cognitive tasks^51–53^, further supporting the idea that dimensional biases are independent of the involved cognitive functions.

### Evidence against the bottom-up processes as the sole origin of the dimensional bias

If dimensional biases merely emerge from advantages in processing of particular object features in early visual pathways, then such advantages should be independent of higher-level cognitive functions, and uniformly enhance the processing of the preferred object feature (dimension). Previously, we examined monkeys and humans in a modified version of the WCST^13^ in which the conflict between the two dimensions (colour and shape) was changed trial-by-trial, and therefore subjects had to resolve competition-conflict between dimensions in congruent (low-conflict) and incongruent (high-conflict) conditions. In both species, the difference in performance between congruent and incongruent conditions (conflict cost) was attenuated in the preferred dimension^13^. If dimensional biases to colour or shape were merely originating in earlier processing stages to enhance a particular object feature, then such processing advantages should have occurred uniformly in both congruent and incongruent conditions, and therefore the conflict cost should have remained the same in both the preferred and non-preferred dimension. Instead, these findings indicate that the dimensional biases were not independent of higher cognitive processes, such as those involved in detecting and resolving conflict^1,23^ and therefore do not confirm the idea that the dimensional biases simply originate in the early visual areas.

## Findings in the current study

### Dimensional biases appear even when matching is done based on the memory of objects

In the context of the modified working memory task, we found that dimensional biases were observed in both humans and monkeys (Fig. 6). Humans demonstrated a bias toward the colour dimension (Fig. 5a,b), while monkeys demonstrated a bias toward the shape dimension (Fig. 2). These findings indicate that dimensional biases appeared in humans' and monkeys' behaviour even when matching was done based on the memory of the objects (i.e. in the absence of actual objects in the visual field). This finding does not conform to the bottom-up scenario, which assumes that dimensional bias is merely dependent on advantages in early visual processing because even in the absence of objects in the visual field, the dimensional biases emerged in behaviour. This conclusion does not mean that dimensional bias is independent of possible mnemonic functions of visual association areas in temporal and parietal regions; instead, it indicates that a simple processing advantage in visual sensory information processing does not explain the dimensional biases in humans and monkeys.

### Dimensional biases show the same direction (species-specific preference) across various cognitive tasks

In macaque monkeys, the dimensional bias in the modified working memory task was toward the shape dimension. This is consistent with the direction of dimensional biases we have observed in the context of the WCST^13^ and the Stop-signal task^7^. In humans, the dimensional bias in the modified working memory task was toward the colour dimension. This is consistent with the direction of dimensional biases in the WCST^13^, in other memory and attention tasks that did not require dimension-shifting^51^, in manual target selection tasks^52^, and two-alternative forced-choice tasks^53^. These findings further support the idea that the neural mechanisms that mediate dimensional bias in monkeys and humans influence a wide range of cognitive functions across various cognitive tasks and emerge earlier in information processing and then influence other cognitive functions.

Previous studies in humans have indicated that dimensional biases in behaviour and eye movements are significantly altered in neurodevelopmental and neuropsychological disorders and are also accompanied by deficits in task performance^13,15–17,19,21,22^. These studies suggest that dimensional biases might be linked to neuropathological processes afflicting these patients. Our findings in this study also indicate that the magnitude of the observed dimensional bias correlated with the task performance (Fig. 7). In both species, the magnitude of dimensional bias in the species-preferred dimension (colour in human, shape in monkey) negatively correlated with accuracy (Fig. 7b). In humans, but not monkeys, there was also a positive correlation between the magnitude of dimensional bias and response time (Fig. 7a). Our findings confirm that in both species, even in healthy subjects, there is a link between dimensional biases and cognitive abilities in resolving the task demands.

### Dimensional biases were influenced by the mnemonic demands of the task

To further examine the mechanisms underlying the emergence of dimensional biases, we examined whether such biases interacted with task difficulty/mnemonic load, which potentially depend on higher-level cognitive processes^37,38^. If a processing advantage in visual pathways solely mediates dimensional biases, they would be independent of higher-level cognitive functions. In monkeys, task difficulty/mnemonic load was altered trial-by-trial by implementing a variable delay (Fig. 1a). The magnitude of task difficulty/mnemonic load influenced monkeys' performance: response time increased (Fig. 3a) and accuracy decreased (Fig. 3b) as the mnemonic load (delay duration) increased. Importantly, the magnitude of such behavioural impairment (delay cost) was modulated by the dimensional bias: delay cost was attenuated in the shape (preferred) dimension (Fig. 4). This indicates that the dimensional bias was not independent of the mnemonic demands of the task, and therefore does not merely arise from simple processing advantages for particular object features.

We have previously observed an interaction between dimensional bias and conflict level in both humans and monkeys, which appeared as a 'smaller conflict cost in the preferred dimension'^13^. In monkeys, the smaller conflict cost was in the shape dimension. Interestingly, in the DMS task in this study, monkeys also showed a smaller delay cost in the shape dimension (Fig. 4). These complementary findings further suggest that dimensional biases interact with other non-sensory and task-specific cognitive functions related to higher cognitive demands and task difficulty.

The significant interaction of dimensional biases with other task-specific cognitive functions suggests that the dimensional biases are not simply a by-product of processing advantages in early visual pathways, and instead depend on neural mechanisms that interact with non-sensory and advanced cognitive functions. In the context of the modified DMS, the dimensional bias might interact with working memory processes^12^ and enhance information processing in the preferred dimension. Previous studies have indicated that modulatory signals from prefrontal and parietal areas have the capacity to selectively enhance the representation of task-relevant information^54–56^, and interact with working memory processes^56–58^. A recent electrophysiological study in monkeys also revealed that a shared neural mechanism might underlie the selection of items from working memory, and the attentional processes related to the perception of sensory stimuli^56^.

### Arousal and autonomic nervous functions were influenced by the dimensional biases

We found that in humans, the dimensional bias also modulated the arousal level and appeared as a significantly lower electrodermal response to the task-related events in the colour (preferred) dimension (Fig. 5c). These changes in electrodermal activity (activity of sweat glands controlled by sympathetic nerve activity) may reflect changes in participants' emotional and arousal state in association with the cognitive difficulty or uncertainty in processing and matching in a specific dimension^13,23,49,59–61^. In both the WCST^13^ and the modified working memory task in this study, the arousal level was lower when humans guided their behaviour based on the colour dimension, suggesting that the task difficulty or uncertainty was presumably lower in the colour (preferred) dimension than in the shape dimension.

### The direction of dimensional bias differs between humans and monkeys

Our findings indicate that humans consistently show bias to colour dimension in the WCST^13^, DMS (Fig. 5), and other cognitive tasks^51–53^; whilst macaque monkeys consistently express significant bias toward shape dimension in the WCST^13^, Stop-signal task^7^, and DMS (Fig. 2). These indicate a systematic behavioural bias to a particular dimension in humans and monkeys, but it is unclear why these biases are in opposite directions. Previous studies indicate that humans and monkeys have comparable visual acuity, trichromatic colour vision, and sensitivity to luminance-contrast of visual stimuli^62–65^. These primate species also show comparable abilities for object recognition^66,67^, perceiving visual illusions^68^, and detection of temporal features of visual stimuli^69^. Neuroanatomical and electrophysiological studies also indicate close homologies in the overall structure and organisation of visual processing and pathways between these species^67,69–71^. Therefore, the opposite biases in humans and monkeys cannot be simply attributed to the differences in detection and perception of colours or shapes, particularly because the same distinct colours and shapes were used in our studies to assess the dimensional biases in humans and monkeys.

The difference in the direction of bias between humans and monkeys might be related to differences in sensitivity and focus toward a particular aspect of visual stimuli or even to the mnemonic representation of those stimuli. Macaque monkeys show more sensitivity to local changes, whereas humans express more consistency in selecting the focus of their attention when they are supposed to covertly or overtly attend to visual stimuli^72,73^. Macaque monkeys focus more on local features than global features of visual stimuli; however, humans show more focused attention on global features^72,74,75^. Such tendency in macaque monkeys to focus on local features of visual objects might lead to more advantageous processing of shape compared to colour information. Conversely, the bias to colour dimension in humans may result from the object colour emerging as a global feature, therefore gaining salience and an advantage in allocation of attentional resources.

## Conclusion

In this study, we have examined crucial questions regarding the emergence of dimensional biases in primate behaviour. Combined with previous findings^7,13^, our results help to further clarify the origin of dimensional biases in primates' cognition. Although there are many unanswered questions regarding the neural substrate and underlying mechanisms of such biases, our findings suggest that the dichotomy of top-down and bottom-up processing^13,33,35,36^ does not fully explain the emergence of dimensional biases. Instead, dimensional biases may emerge when processed information regarding visual object features interface with non-sensory processes (such as mnemonic and executive functions) to guide goal-directed behaviour. We still do not know why such dimensional biases are evolutionarily preserved in the behaviour of two primate species. It is also unknown why dimensional biases are in opposite directions in humans and monkeys, and whether the direction of dimensional bias in apes (such as the bonobo and chimpanzee) is similar to that in humans or macaque monkeys. Considering that dimensional biases are altered in certain neurodevelopmental and neuropsychological disorders^13,15–17,19,21,22^, delineating the significance of dimensional biases and their underlying neural mechanisms might also help gain insight into many unknown questions regarding the neurobiological basis of such disorders.

## Data Availability

Data is available upon reasonable request to the corresponding author.
